# *In Vivo* Delivery of Gremlin siRNA Plasmid Reveals Therapeutic Potential against Diabetic Nephropathy by Recovering Bone Morphogenetic Protein-7

**DOI:** 10.1371/journal.pone.0011709

**Published:** 2010-07-22

**Authors:** Qingxian Zhang, Yonghong Shi, Jun Wada, Sandra M. Malakauskas, Maodong Liu, Yunzhuo Ren, Chunyang Du, Huijun Duan, Yingmin Li, Ying Li, Yanling Zhang

**Affiliations:** 1 Department of Nephrology, Third Hospital, Hebei Medical University, Shijiazhuang, China; 2 Department of Pathology, Hebei Medical University, Shijiazhuang, China; 3 Department of Medicine and Clinical Science, Okayama University Graduate School of Medicine, Okayama, Japan; 4 Division of Nephrology, University of Alabama at Birmingham, Birmingham, Alabama, United States of America; University of Tor Vergata, Italy

## Abstract

Diabetic nephropathy is a complex and poorly understood disease process, and our current treatment options are limited. It remains critical, then, to identify novel therapeutic targets. Recently, a developmental protein and one of the bone morphogenetic protein antagonists, Gremlin, has emerged as a novel modulator of diabetic nephropathy. The high expression and strong co-localization with transforming growth factor- β1 in diabetic kidneys suggests a role for Gremlin in the pathogenesis of diabetic nephropathy. We have constructed a gremlin siRNA plasmid and have examined the effect of Gremlin inhibition on the progression of diabetic nephropathy in a mouse model. CD-1 mice underwent uninephrectomy and STZ treatment prior to receiving weekly injections of the plasmid. Inhibition of Gremlin alleviated proteinuria and renal collagen IV accumulation 12 weeks after the STZ injection and inhibited renal cell proliferation and apoptosis. *In vitro* experiments, using mouse mesangial cells, revealed that the transfect ion of gremlin siRNA plasmid reversed high glucose induced abnormalities, such as increased cell proliferation and apoptosis and increased collagen IV production. The decreased matrix metalloprotease level was partially normalized by transfection with gremlin siRNA plasmid. Additionally, we observed recovery of bone morphogenetic protein-7 signaling activity, evidenced by increases in phosphorylated Smad 5 protein levels. We conclude that inhibition of Gremlin exerts beneficial effects on the diabetic kidney mainly through maintenance of BMP-7 activity and that Gremlin may serve as a novel therapeutic target in the management of diabetic nephropathy.

## Introduction

Diabetic nephropathy (DN) is the leading cause of end-stage renal disease and about 20% to 40% of patients with diabetes ultimately develop diabetic nephropathy[1], [2]. Specific therapies to reverse or inhibit the progression of diabetic nephropathy to advanced stages are not available and current treatment strategies are limited to management of blood glucose levels and control of hypertension[3], [4]. Diabetic nephropathy is characterized by various pathological features, such as renal cell proliferation and apoptosis, mesangial expansion and sclerosis, glomerular basement membrane thickening and the subsequent development of tubulointerstitial fibrosis[2]. Hyperglycemia is the major factor precipitating renal injury in this setting[5]. However, the downstream signaling pathways which influence this process are not fully defined. One known mediator in the development of both glomerulosclerosis and tubulointerstitial fibrosis is transforming growth factor- β1 (TGF-β1)[6]; however, because of its pleiotropic actions, TGF-β may not be an ideal therapeutic target. Recently, a role for the re-activation of developmental programs in DN has been recognized[7]. Increased gene expression of such molecules as connective tissue growth factor (CTGF), vascular endothelial growth factor (VEGF), bone morphogenetic proteins (BMPs) and gremlin, a BMP antagonist, supports the notion that ontogenic processes are operative in the development of DN[6], [8], [9], [10].

Gremlin is a 184-amino acid protein which is present in both soluble and cell-associated forms. It is highly conserved and is a member of the structural cysteine knot superfamily. Functionally, Gremlin plays an important role in development and belongs to a novel family of bone morphogenetic protein (BMP) antagonists that include the head inducing factor Cerberus and the tumor suppressor DAN[11]. Under basal conditions, Gremlin is present at relatively low levels in the adult kidney[12], [13]. However, it is highly expressed in biopsy specimens from patients with diabetic nephropathy, where it is predominantly observed in areas of tubulointerstitial fibrosis and where it co-localizes with TGF-β1 expression[12], [13]. In addition, Gremlin mRNA levels correlate directly with elevated serum creatinine levels and tubulointerstitial fibrosis scores in patients with DN[12]. Further, Gremlin expression is enhanced in mesangial cells cultured under high glucose conditions and in those exposed to cyclic mechanical strain and transforming growth factor-β (TGF-β)[7]. Collectively, these data suggest a role for Gremlin in the pathogenesis of tubulointerstitial fibrosis in DN. Thus, we hypothesize that Gremlin may serve as a therapeutic target in the management of this disease. To explore this possibility, we utilized a mouse model of diabetic nephropathy (uninephrectomy and streptozotocin (STZ) treatment) to examine the effect of siRNA-induced Gremlin inhibition *in vivo* on the progression of renal pathology.

## Results

### Gremlin Expression in Mouse Kidney is Inhibited by Gremlin siRNA Plasmid

**Figure 1:**
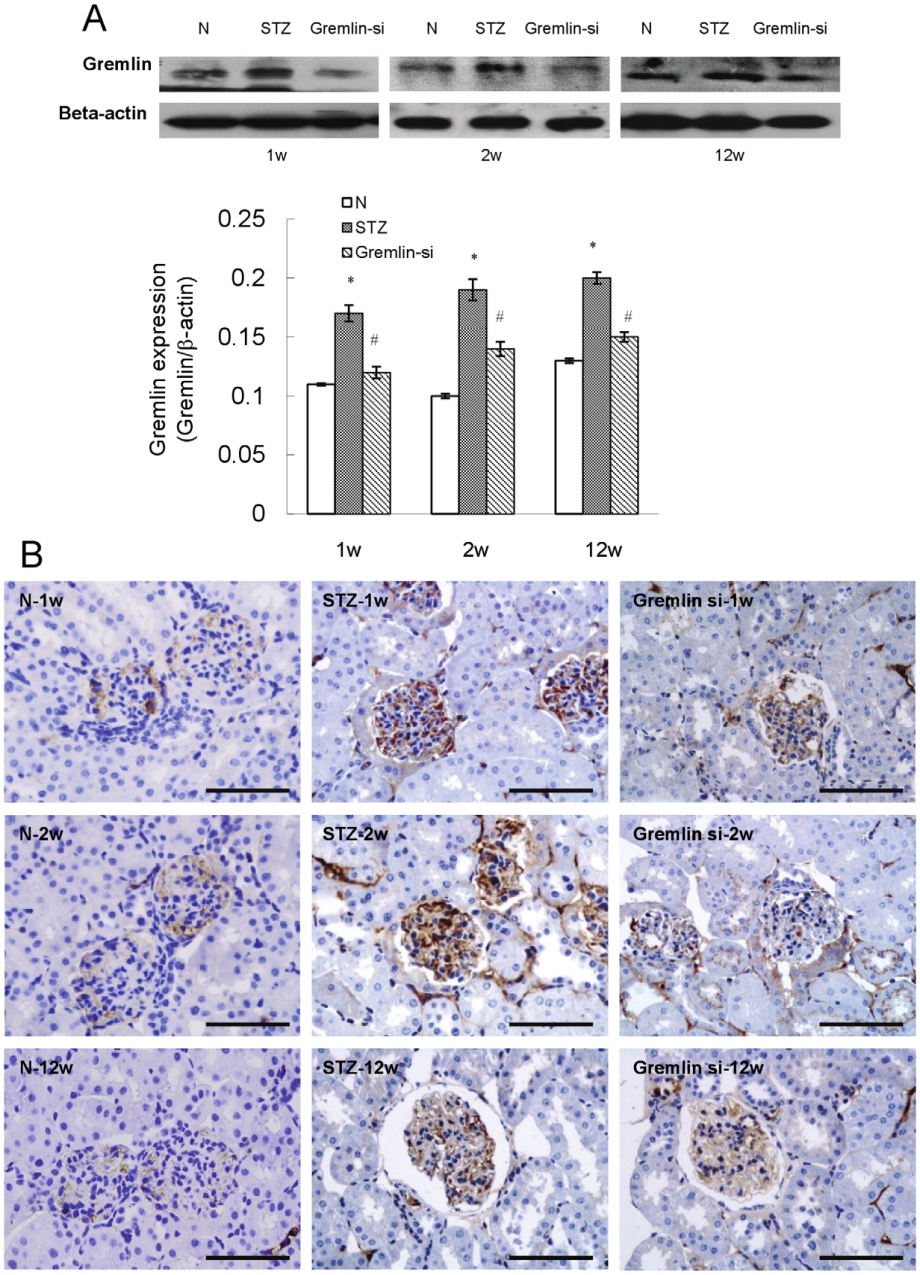
Delivery of gremlin siRNA plasmid into diabetic CD-1 mice post-uninephrectomy. (**A**) Gremlin protein expression by western blotting in whole-kidney homogenates at different time points after injection of pBAsi mU6 Neo control vector or pBAsi mU6 Neo gremlin siRNA plasmid, respectively. Compared to those treated with pBAsi mU6 Neo plasmid (STZ group), animals administered pBAsi mU6 Neo gremlin siRNA plasmid (Gremlin siRNA group) show low expression of Gremlin in the kidneys. (**B**) Immunostaining of kidney sections shows the localization of Gremlin protein after the delivery of plasmids. Marked Gremlin expression is observed in both glomeruli and tubules in the STZ group, which is significantly inhibited by the delivery of gremlin siRNA plasmid. (* p<0.01 vs. non-diabetic control group; #p<0.05 vs. STZ group). Scale bars, 100 µm. N = 6 mice per group.

As seen in **Figure 1A**, Gremlin protein expression in the STZ-treated group was about 1.5-fold greater than in the non-diabetic control mice (N). Treatment with gremlin siRNA plasmid significantly inhibited Gremlin expression induced by diabetic conditions (Gremlin-si). Immunostaining (**Figure 1B**) revealed that, in the non-diabetic control group, Gremlin expression was predominantly detected in glomeruli, while signal was barely seen in tubules and interstitial areas. In the STZ group, Gremlin was highly expressed in glomeruli and also in interstitial areas and part of tubules at week-2. In the Gremlin-si group, Gremlin expression was significantly weaker in both glomeruli and tubular interstitial areas, indicating a successful inhibition of Gremlin expression by gremlin siRNA plasmid (**Figure 1B**).

### Treatment with Gremlin siRNA Plasmid Alleviates Proteinuria, Serum Creatinine Elevation and Renal Hypertrophy

**Figure 2:**
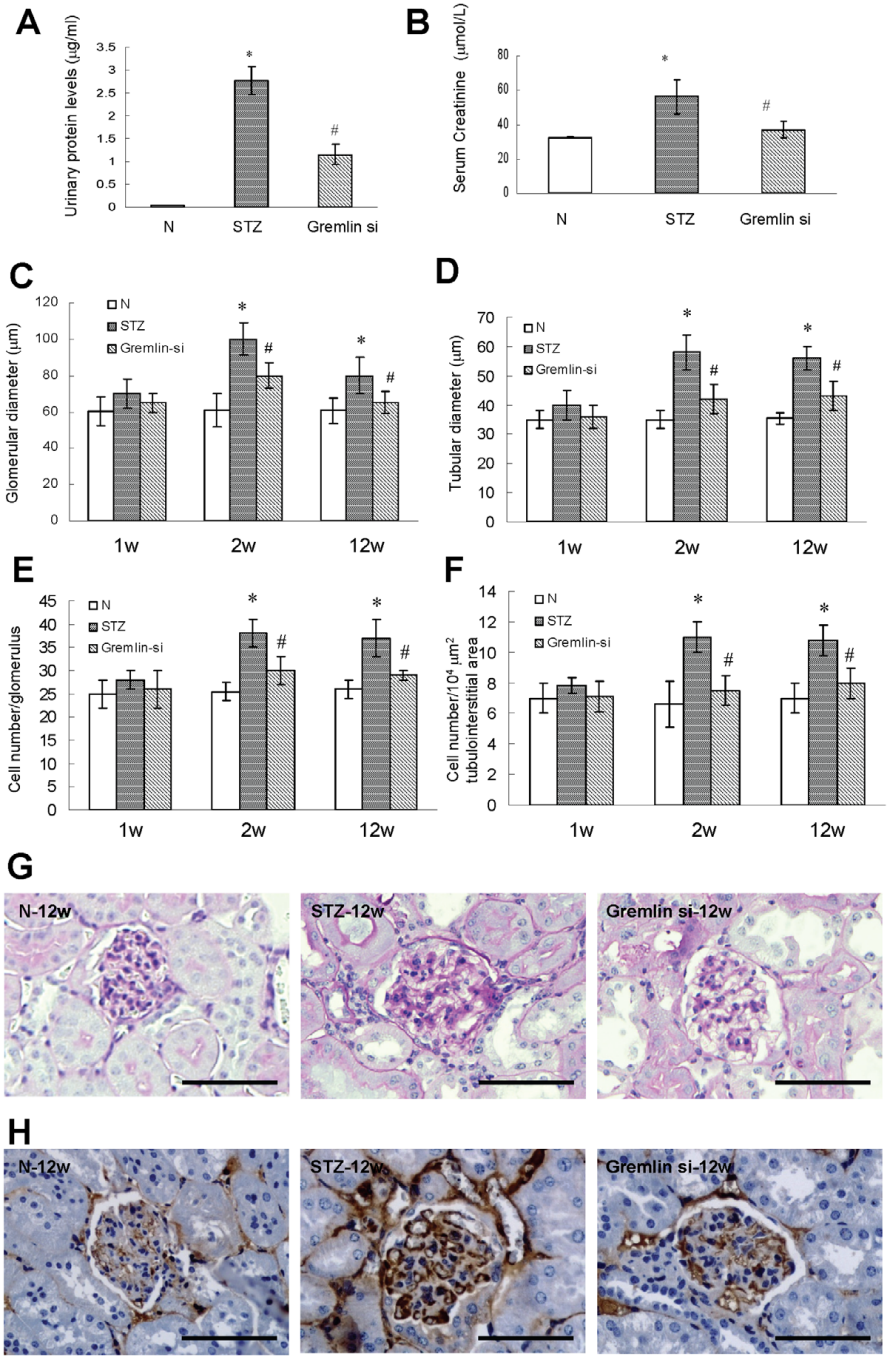
The effects of gremlin siRNA plasmid delivery on diabetic nephropathy in diabetic mice post-uninephrectomy. (**A**) Increased spot urinary protein levels and (**B**) serum creatinine in STZ-induced diabetic mice treated with pBAsi mU6 Neo control plasmid (STZ) compared with nondiabetic control animals (N). The effect is decreased by treatment of diabetic animals with pBAsi mU6 Neo gremlin siRNA plasmid (Gremlin-si). (**C, D**) Increase in glomerular and tubular diameters at week 2 and week 12 is ameliorated by treatment with gremlin siRNA plasmid. (**E, F**) Increases in cell numbers in both glomeruli and tubules at week 2 and week 12 are significantly reduced in the Gremlin siRNA group. (**G**) PAS staining of kidney tissues shows glomerular and tubular hypertrophy and mesangial matrix accumulation in the STZ group 12 weeks after STZ injection. Treatment with gremlin siRNA plasmid prevents these pathological changes. (**H**) Collagen type IV expression in the kidneys at week 12. High expression of collagen IV is seen in diabetic kidney and the treatment of gremlin siRNA plasmid significantly down-regulated the accumulation of collagen IV. (* p<0.05, vs. non-diabetic control group, ^#^ p<0.05, vs. STZ group). Scale bars, 100 µm (**G, H**). N = 6 mice per group.

At week-12, the urinary protein level was dramatically higher in the STZ group compared to control. Gremlin siRNA plasmid treatment significantly reduced proteinuria (**Figure 2A**). The serum creatinine was also increased in the STZ group compared with that of control, and treatment with gremlin siRNA plasmid significantly reduced the high level of serum creatinine in diabetic mice (**Figure 2B**). In addition, the glomerular and tubular diameters and cell numbers significantly increased in the STZ group compared with those of the control mice, while the treatment with gremlin siRNA plasmid alleviated these changes (**Figure 2, C, D, E & F**). We further investigated the protective effects of treatment with gremlin siRNA plasmid on diabetic nephropathy by assessment of the histopathological changes and collagen type IV accumulation at week-12. Diabetic mice in the STZ group exhibited significant tubular and glomerular hypertrophy, widened mesangial areas, as well as increased collagen type IV expression compared with the non-diabetic control group. Treatment with gremlin siRNA plasmid was associated with a significant reduction in renal hypertrophy, mesangial areas and accumulation of collagen type IV (**Figure 2G, H**). These data demonstrate that gremlin siRNA plasmid delivery significantly inhibited glomerular and tubular hypertrophy in diabetic kidneys from week 1 to week 12, alleviated proteinuria and displayed a protective effect on renal function at week 12.

### Delivery of Gremlin siRNA Plasmid Inhibits Renal Cell Proliferation and Apoptosis in Diabetic Mice

**Figure 3:**
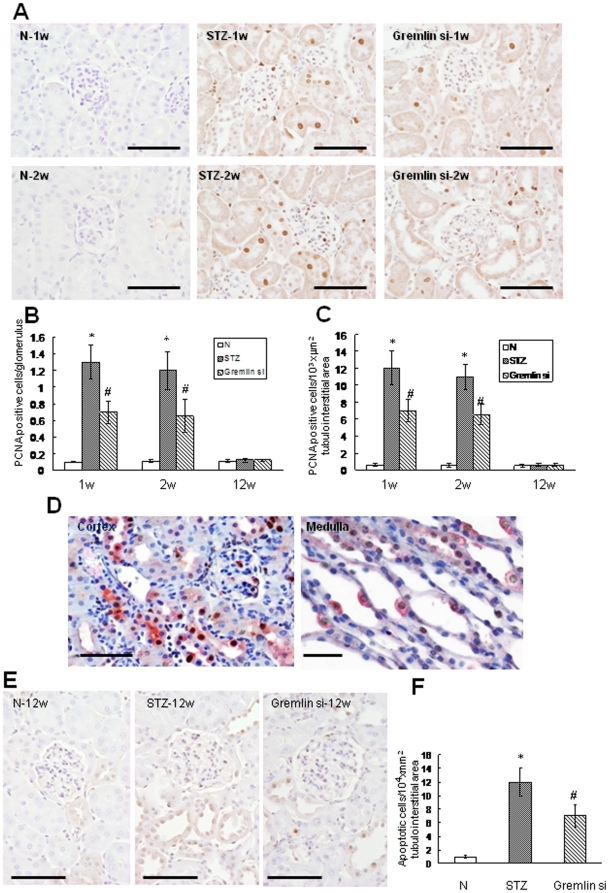
Cell proliferation and apoptosis in diabetic mouse kidneys. (**A**) Detection of proliferating cell nuclear antigen (PCNA) by immunoperoxidase staining, in the kidneys of non-diabetic control mice (N), streptozotocin-induced diabetic mice treated with pBAsi mU6 Neo control plasmid (STZ) or pBAsi mU6 Neo gremlin siRNA plasmid (Gremlin siRNA). (**B** and **C**) PCNA positive cells in kidneys from the STZ group dramatically increase at week-1 and -2, and pBAsi mU6 Neo gremlin siRNA plasmid treatment significantly reduces PCNA positive cells both in glomeruli and tubules. Proliferating cells are barely seen in all three groups at week 12. (**D**) Co-immunostaining of diabetic kidney sections with antibodies against PCNA and Gremlin. Intensive Gremlin expression is often seen in the cells with PCNA positive signal. (**E, F**) *In situ* TUNEL assay. Apoptotic cells are observed predominantly in tubules in the STZ group at week-12. The number of apoptotic cells is significantly reduced by pBAsi mU6 Neo gremlin siRNA plasmid treatment. (* p<0.01 vs. non-diabetic control group, # p<0.01 vs. STZ group). Scale bars, 100 µm (**A, B** and **E**), and 10 µm (**D**). N = 6 mice per group.

Proliferation of kidney cells was evaluated with PCNA staining. PCNA positive cells were occasionally seen in the non-diabetic control group and were significantly increased in the tubules and glomeruli of the STZ group at week-1 and -2. Delivery of gremlin siRNA plasmid reduced the numbers of PCNA positive cells. By week-12, the numbers of PCNA positive cells returned to basal levels in the STZ and Gremlin-si groups, and there were no differences among the three groups (**Figure 3, A, B & C**). The kidney tissue of the diabetic mice at week-2 was double stained with antibodies against PCNA and Gremlin. PCNA positive signals were often seen in cells with intense Gremlin expression, both in glomeruli and tubules, as well as in the renal medulla (**Figure 3D**). No apparent apoptotic cells were seen in the three groups at week-1 and week-2; at week-12, cell apoptosis was barely seen in the non-diabetic control group and in glomeruli from the STZ group. However, there was clustering of apoptotic cells in the tubules of the STZ group. Treatment with gremlin siRNA plasmid significantly reduced the number of apoptotic cells (**Figure 3E, F**).

### Expression of BMP-7 in Diabetic Kidney is not directly Regulated by Gremlin

**Figure 4:**
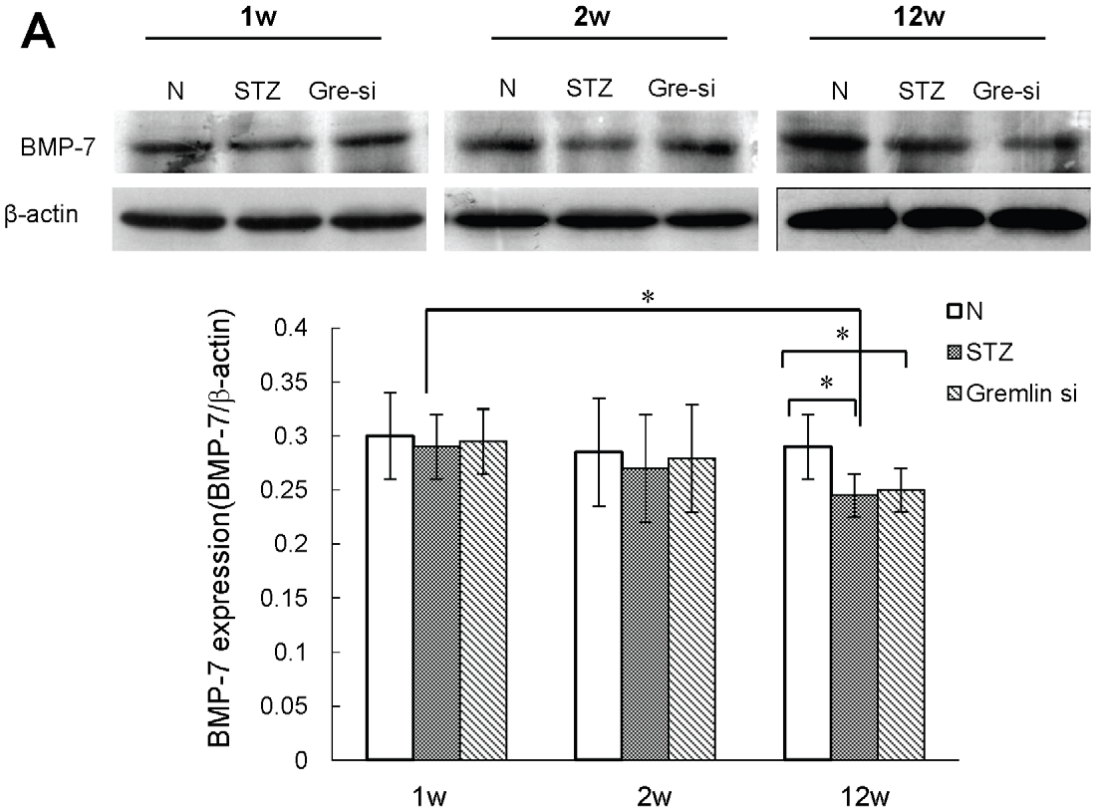
BMP-7 expression in diabetic kidneys assessed by Western blotting. Compared with non-diabetic control mice (N), mice in the STZ group display similar BMP-7 kidney expression levels at week-1 and week-2. The BMP-7 expression in the STZ group gradually decreased to a significantly lower level at week-12. No significant effect is seen on the expression of BMP-7 in diabetic kidneys by the treatment with gremlin siRNA plasmid. (* p<0.05). N = 6 mice per group.

As shown in **Figure 4**, expression of BMP-7 in kidney cortical homogenates from the STZ group markedly decreased compared to that of the control group at week-12. No obvious effect of gremlin siRNA plasmid on BMP-7 expression in the diabetic kidney was seen, which indicated that BMP-7 expression in the kidneys of STZ-induced diabetic rats may not be directly regulated by Gremlin.

### Transfection with Gremlin siRNA Plasmid Normalizes Cell Proliferation Induced by Exposure to High Glucose Levels

**Figure 5:**
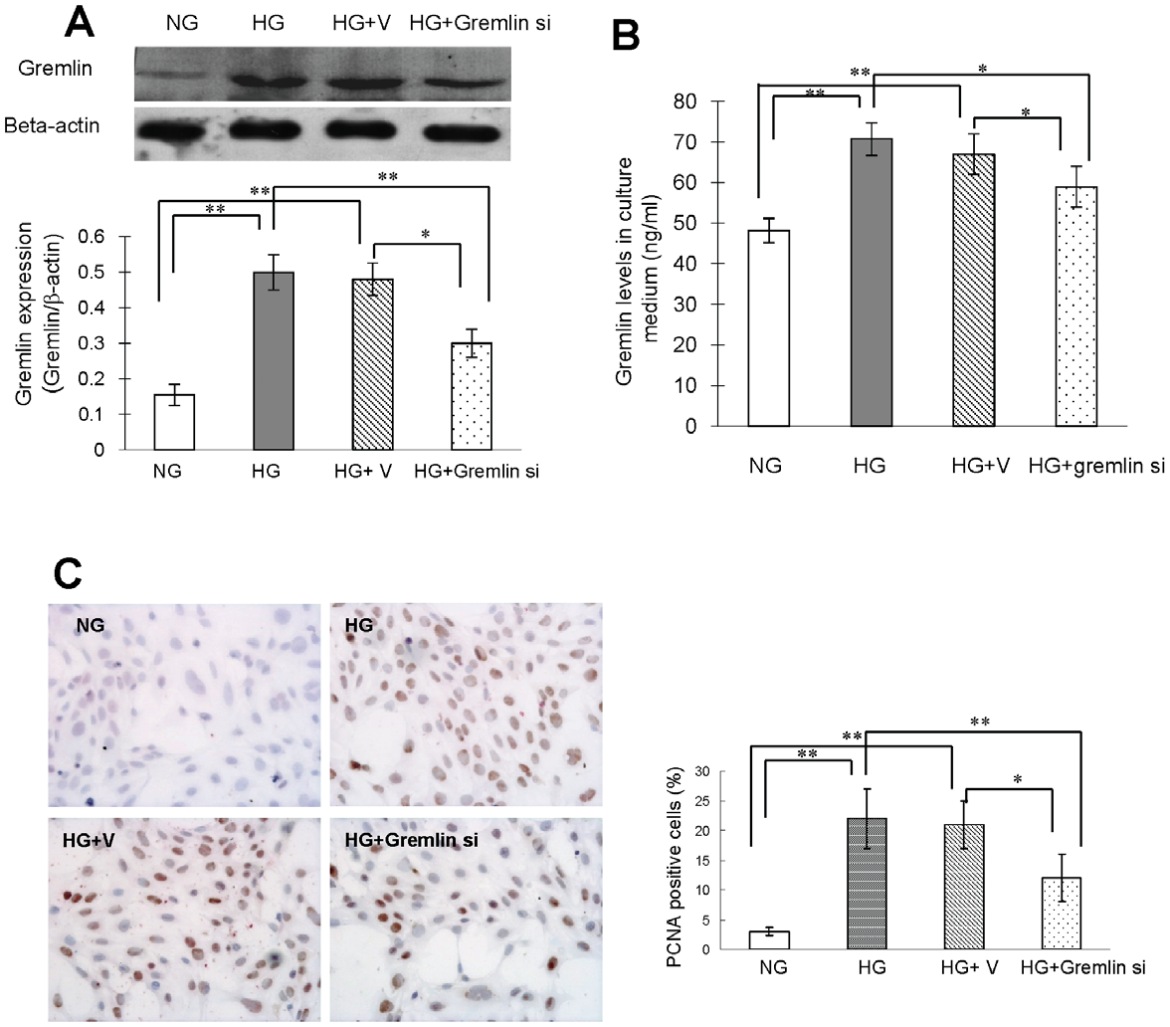
Cell proliferation in human mesangial cells cultured under high glucose conditions. Human mesangial cells were cultured in RPMI 1640 containing normal glucose (100 mg/dl D-glucose; NG) and high glucose (300 mg/dl D-glucose; HG). Cells under HG conditions were transfected with pBAsi mU6 Neo control plasmid (HG+V) or pBAsi mU6 Neo gremlin siRNA plasmid (HG+gremlin si) 12 hours before the glucose stimulation. Cell proliferation was examined by PCNA staining 12 hours after glucose stimulation. Gremlin expression is examined in human mesangial cells by Western blot (**A**); the secreted Gremlin in culture medium is observed by ELISA (**B**). The HG stimulated Gremlin expression in human mesangial cells is successfully inhibited by the transfection of pBAsi mU6 Neo gremlin siRNA plasmid. (**C**) High glucose-induced cell proliferation is inhibited in the HG+gremlin si group. (* p<0.05, ** p<0.01). Six independent experiments were repeated.

Mouse mesangial cells were transfected with control or gremlin siRNA plasmid and then assessed for cell proliferation by PCNA staining after high glucose (HG) stimulation. Gremlin protein expression was efficiently inhibited by transfection with gremlin siRNA plasmid, as demonstrated by Western blot analysis of cell extracts (**Figure 5A**) and by ELISA using culture medium (**Figure 5B**). As shown in **Figure 5C**, the number of proliferative cells significantly increased in the HG group (21±5%) and the HG and control plasmid group (20±4%). Transfection with gremlin siRNA plasmid into MCs significantly inhibited the HG-induced cell proliferation (12±4%).

### Transfection with Gremlin siRNA Plasmid Reduces Collagen Type IV Accumulation in Cells Exposed to High Glucose

**Figure 6:**
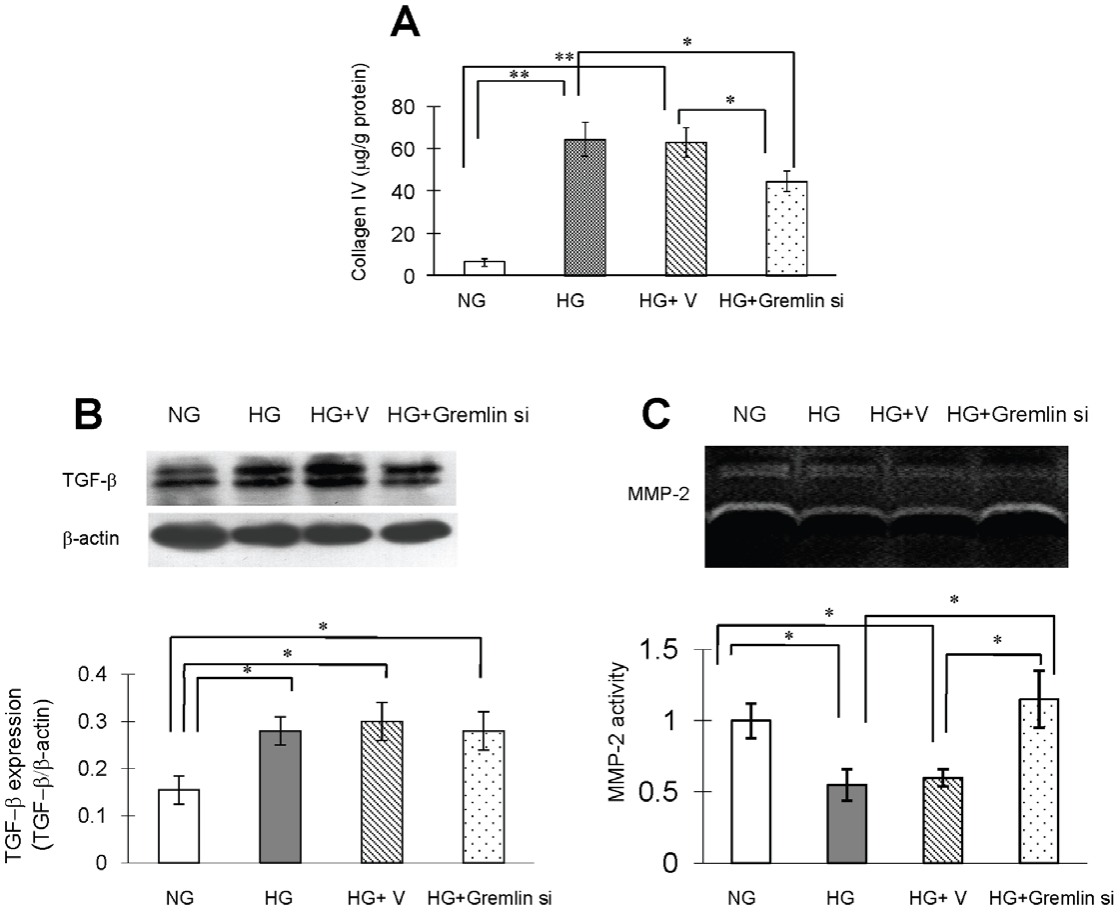
Collagen type IVand TGF-βexpression and MMP-2 activity in mouse mesangial cells cultured under high glucose conditions. Mouse mesangial cells were cultured in RPMI 1640 and transfected with pBAsi mU6 Neo or pBAsi mU6 Neo gremlin siRNA plasmid as described in the methods. Culture medium was collected for measurement of collagen type IV concentration by radio-immunoassay, and cells were subjected to Western blot analysis to determine MMP-2 and TGF-βexpression levels 48 hours after glucose stimulation. (**A**) Increased collagen type IV accumulation is observed in the HG group, and gremlin siRNA plasmid transfection significantly inhibits collagen type IV secretion. (**B**) Compared to the normal glucose control group (NG), TGF-β expression is significantly increased under high glucose conditions, and the HG stimulated TGF-β expression remains the same after gremlin siRNA transfection. (**C**) Compared with the NG group, MMP-2 activity in culture medium is significantly decreased in the HG and HG+V groups, and this is prevented by transfection with gremlin siRNA plasmid. (* p<0.05, ** p<0.01). Six independent experiments were repeated.

To evaluate the influence of Gremlin inhibition on collagen type IV synthesis and possible mechanisms of interaction, cultured mouse mesangial cells were again transfected with control or gremlin siRNA plasmid and then subjected to stimulation with high glucose. Collagen type IV levels in the culture medium were determined by radio-immunoassay, and cells were collected for Western blot analysis of TGF-β, and matrix metalloprotease-2 (MMP-2) activity in culture medium was determined by zymography (**Figure 6**). Significant accumulation of collagen type IV in the culture medium was seen in the HG and HG+V groups, while gremlin siRNA plasmid transfection significantly reduced the collagen type IV accumulation (**Figure 6A**). TGF-β expression significantly increased under high glucose conditions, and no obvious effect was observed after gremlin siRNA transfection. On the other hand, MMP-2 activity was significantly decreased in the HG and HG+V groups compared to control. The glucose-induced suppression of MMP-2 activity was inhibited by transfection with gremlin siRNA plasmid (**Figure 6B**).

### Gremlin Interacts with BMP-7 and Regulates BMP-7 Activity in Mesangial Cells

**Figure 7:**
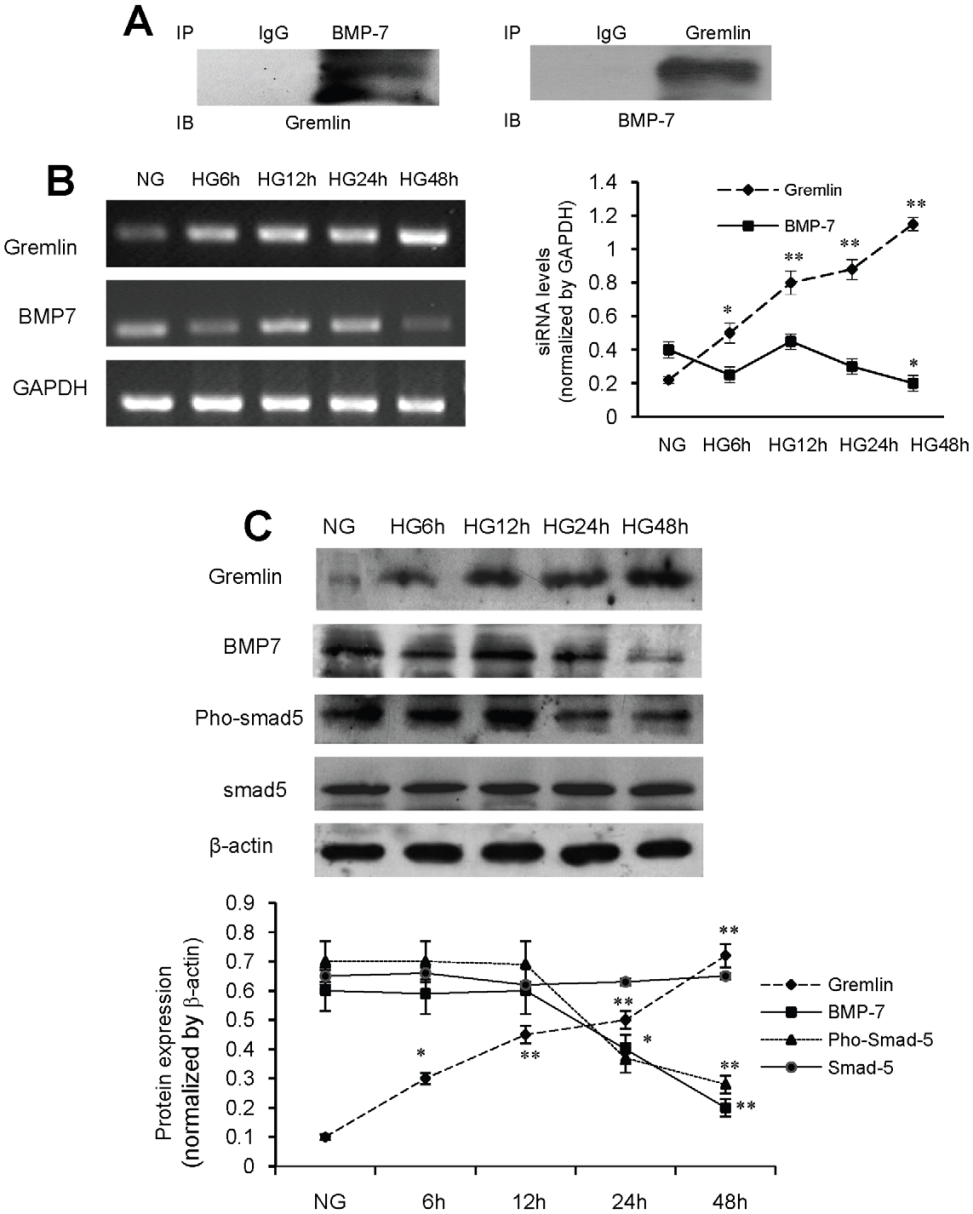
Gremlin interacts with BMP-7 and regulates BMP-7 activity in mesangial cells. Mouse mesangial cells were cultured in RPMI 1640 and collected 6 h, 12 h, 24 h and 48 h after HG stimulation. (**A**) Co-immunoprecipitation demonstrates an interaction between BMP-7 and Gremlin in mesangial cells. (**B**) mRNA levels of gremlin and BMP-7 are detected by RT-PCR. After HG stimulation, a significant increase in Gremlin mRNA level is observed after 6 hours incubation in high glucose, and the expression gradually increases with the culture duration. (**C**) The expression of BMP-7 mRNA dramatically decreases 48 hours later. Accordingly, increased Gremlin protein levels are observed in the cultured cells. Corresponding to a decrease in the protein level of BMP-7, the level of Smad-5 remained constant, whereas phosphorylated Smad-5 significantly and gradually decreases from 12 h to 48 h (* p<0.05, ** p<0.01 vs. the value of NG group).

**Figure 8:**
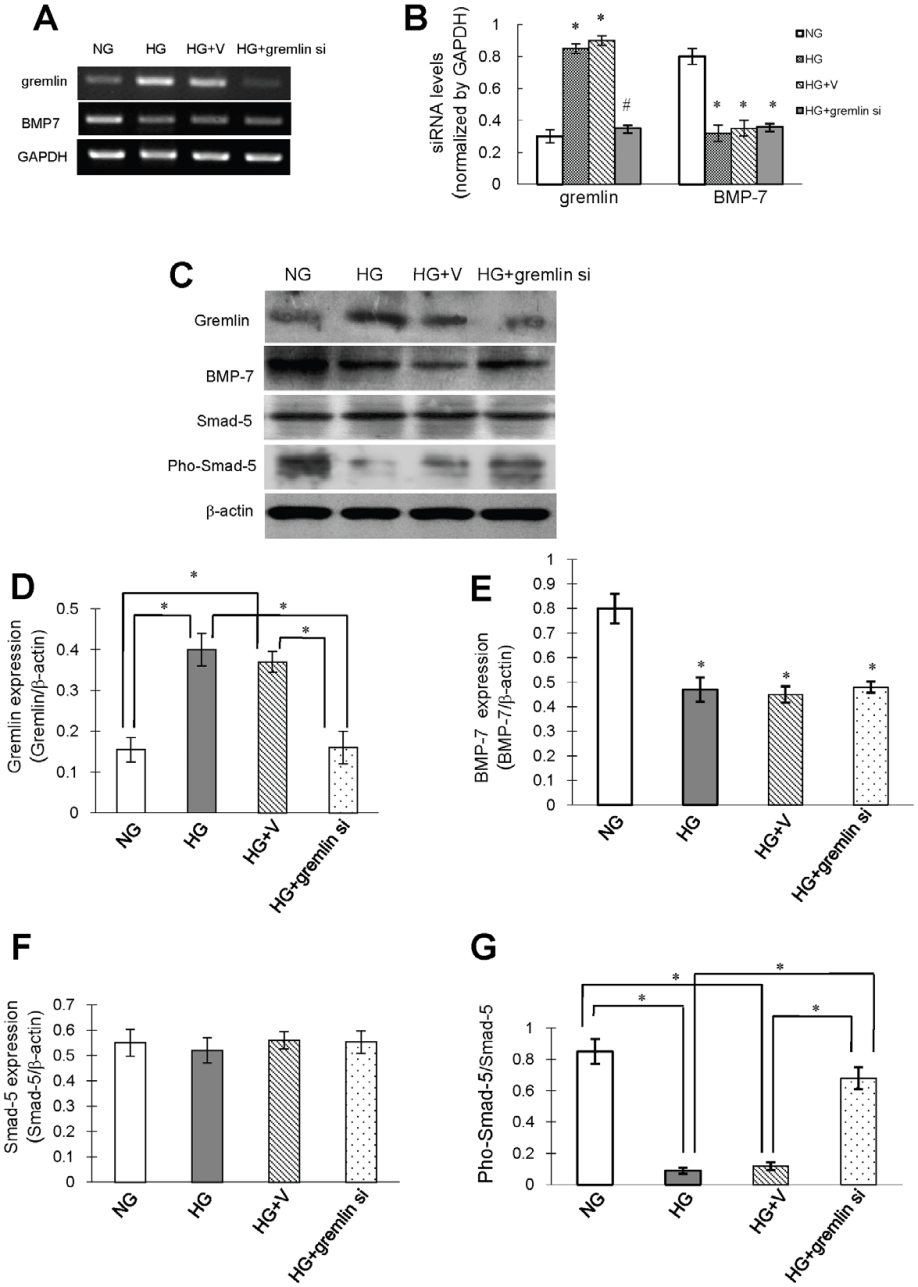
BMP-7 activity in mouse mesangial cells transfected with gremlin siRNA plasmid. Mouse mesangial cells were transfected with pBAsi mU6 Neo or pBAsi mU6 Neo gremlin siRNA plasmid and stimulated with NG and HG. Cells were collected 48 hours after HG stimulation and subjected to RT-PCR and Western blot. BMP-7 mRNA level was found decreased after gremlin siRNA transfection (**A & B**). The protein levels of BMP-7 and Phos-Smad-5/Smad-5 decreased after 48 hours incubation with high glucose. Transfection with gremlin siRNA plasmid significantly increased the Phos-Smad-5/Smad-5 level (* p<0.01), whereas levels of BMP-7 and Smad-5 remained similar (**C, D, E, F, and G**). Six independent experiments were repeated.

To investigate the mechanism by which the inhibition of gremlin expression mediates its renal protective effects, the expression and activity of BMP-7 was examined in mouse mesangial cells cultured under HG conditions. Co-immunoprecipitation revealed a physical interaction between BMP-7 and Gremlin (**Figure 7A**). Incubation of cultured cells under HG conditions over 48 hours revealed a gradual increase in Gremlin expression with associated decrease in BMP-7 at both the mRNA level and protein level (**Figure 7B & C**). Similarly the level of phosphorylated Smad-5, a marker of BMP-7 activity, significantly and gradually went down while total Smad-5 protein levels remained constant (**Figure 7C**). No significant changes in BMP-7 expression were seen after transfection of cells with gremlin siRNA plasmid (**Figure 8A, B, C & E**), whereas the decrease in phosphorylation of Smad-5 was prevented by gremlin siRNA plasmid transfection (**Figure 8C & G**). These results suggest that the protective effects of siRNA-induced inhibition of gremlin expression on DN were, at least partially, through the recovery of BMP-7 activity.

## Discussion

The molecular pathogenesis of diabetic nephropathy has not been fully characterized, and novel mediators of the disease are still being described. The re-activation of developmental programs in DN has shed light on novel pathways influencing the disease and suggests new potential therapeutic targets. Bone morphogenetic proteins, active in development, are homodimeric members of the TGF-βsuperfamily of cysteine-knot cytokines[14], [15]. The TGF-β superfamily comprises over twenty BMPs, of which BMP-7 is the most prominent member involved in renal development and disease. In the adult life, BMP-7 is primarily expressed in kidney tubules, as well as glomeruli[16], [17], [18]. Loss of endogenous BMP-7 expression occurs in diabetic rats and is associated with profibrotic activity[19], [20]. In the streptozotocin diabetic model BMP-7 is reduced by 50% at 15 weeks and continues to decline further to 10% by 30 weeks[21]. In cultured tubular cells, TGF-βdecreases BMP-7 expression, which suggests that a rise in tubular TGF-β levels during the evolution of diabetic nephropathy contributes causally to the loss of BMP7 and BMP7 type I and II receptors. Morrissey and associates showed that exogenously administered recombinant human (rh) BMP-7 may even resolve, at least partially, glomerular and interstitial fibrosis in experimental diabetic nephropathy. BMP-7 activity in the kidney is not only determined by availability of BMP-7 itself, but also by a balance of agonists, such as Kielin/chordin-like protein (KCP) or BMP receptors, and antagonists, such as gremlin, noggin, or uterine sensitization-associated gene-1 (USAG-1), that prohibit BMPs from binding to their cognate receptors[22]. Among three BMP antagonists, only gremlin increases in diabetic rat kidneys[19]. Here we propose that inhibition of Gremlin may induce therapeutic effects on the diabetic kidney by allowing the efficient binding of endogenous BMP-7 to receptors without inhibition.

In the current study, diabetes was induced in CD-1 mice and siRNA plasmid was transferred weekly into the diabetic mice to inhibit Gremlin expression. AMDCC investigators indicate significant diversity among individual CD-1 mice in the levels of albuminuria with low dose STZ diabetes (http://www.amdcc.org/shared/showFile.aspx?doctypeid=7&docid=530), but high dose STZ results in severe diabetic nephropathy in CD-1 mice, which was reported to mimic human diabetic nephropathy in histopathologic lesions and renal function[23], so we used high dose STZ to induce diabetes in CD-1 mice, similar levels in renal function parameters and histological changes were seen in animals within the same experimental group. Our data demonstrate that administration of gremlin siRNA plasmid to diabetic mice alleviated renal hypertrophy, cell proliferation and apoptosis, and subsequently suppressed collagen type IV accumulation and mesangial expansion, indicating beneficial effects of Gremlin inhibition on diabetic nephropathy. A significant reduction in BMP-7 expression at the late stage of diabetic nephropathy has been reported[20]. Based upon our data, the expression level of BMP-7 dramatically dropped to half of the control level by week 12. Gremlin siRNA treatment showed no effect on the reduced expression level of BMP-7 in diabetic kidneys. However, a physical interaction between BMP-7 and Gremlin was demonstrated by immunoprecipitation, and phosphorylated Smad-5, a marker of BMP-7 activity, was up-regulated by gremlin siRNA plasmid transfection. BMPs binding to their receptors activate mothers against decapentaplegic (Smad) signaling, which is revealed by the phosphorylation of Smads[24], [25]. Smad 1, 5 and 8 are receptor regulated Smads (R-Smads) that can be activated by BMPs. Smad5 was found to be the preferred BMP-7-induced receptor-activated Smad signal in kidney[26]. Loss of BMP-7 signaling activity, as illustrated by lower phosphorylated Smad 5 protein level, was observed in experimental diabetic nephropathy[27]. Our results from mesangial cells cultured under high glucose conditions, demonstrate that a gradual increase in Gremlin protein levels from 6 h to 48 h after HG stimulation is associated with decreasing levels of phosphorylated Smad-5. Transfection of these cells with gremlin siRNA plasmid resulted in significantly increased levels of phosphorylated Smad-5, whereas, there was no significant increase of BMP7 level after trasfection of gremlin siRNA plasmid. Taken together, our *in vivo* and *in vitro* data, as well as the functional studies relating to BMP-7 and gremlin reported in the literature, support a model in which the major mechanism of therapeutic action of gremlin inhibition on DN is related to the recovery of BMP-7 activity. Firstly, BMP-7 is involved in ameliorating renal damage due to mesangial proliferation by suppression of mesangial cell mitosis via Smad1, −5, −8 signaling[28]. BMP-7 is also able to prevent metanephric mesenchymal cells and renal epithelial cells from undergoing apoptosis, thereby preserving renal function[29], [30]. From our study, the inhibition of gremlin expression was able to normalize renal cell growth, including HG-induced proliferation and apoptosis. Accumulating evidence suggests that early renal hypertrophy, partially resulting from cell proliferation, acts as a pacemaker for subsequent irreversible structural changes, such as glomerulosclerosis and tubulointerstitial fibrosis[31]. Secondly, maintenance of BMP-7 activity by inhibition of Gremlin expression may result in blockade of extracellular matrix (ECM) accumulation. It was reported that BMP-7 could reduce TGF-β-induced ECM protein accumulation in cultured mesangial cells by maintaining the levels and activity of MMP2, partially through prevention of TGF-β-dependent upregulation of PAI-1[31], [32], [33]. Our data showed that treatment with gremlin siRNA plasmid resulted in a significant reduction in mesangial areas and accumulation of collagen type IV in diabetic mice, and the reduced matrix metalloprotease (MMP-2) level in mesangial cells cultured under HG conditions was enhanced by transfection with gremlin siRNA plasmid.

A specific question should be addressed whether Gremlin has BMP-7-independent effects on the pathogenesis of diabetic nephropathy. As shown in **Figure 3D**, the proliferative activity of mesangial cells is associated with the expression level of Gremlin. It was reported that Gremlin can increase DNA synthesis and cell counts and accelerate cell cycle progression of vascular smooth muscle cells (VSMC) through mechanisms that include p27(kip1) down-regulation[15]. Gremlin was also found overexpressed in various human tumors and widely expressed by cancer-associated stromal cells, and can promote tumor cell proliferation [34], [35], suggesting the ability of proliferation stimulation. Thus it is possible that Gremlin regulates cell growth via a BMP-7-independent pathway.

Overexpression of Gremlin in diabetic kidneys suggests a role for the re-activation of developmental programs in DN. In addition to Gremlin, some other developmental genes, such as FMN1[36], a gene with a Gremlin transcriptional enhancer within the 3′ end of its locus should be considered as well. While Gremlin expression may be regulated by FMN1, knockdown of Gremlin by siRNA plasmid might not affect the expression and function of FMN1.To date, no evidence suggests that Gremlin regulates Fmn1. Thus FMN1 was not measured in the current study. Based on the fact that both Gremlin and FMN1 have important implications for renal system, and the role of FMN1 in gremlin transcriptional regulation, it would be very interesting to investigate whether FMN1 are also associated with diabetic nephropathy in the future study.

In summary, in addition to advancing our knowledge of the pathophysiology of diabetic nephropathy, our data using *in vivo* delivery of gremlin siRNA plasmid has special relevance to new therapies that target Gremlin. Our findings suggest a role for siRNA-mediated gremlin inhibition in protecting the kidney from the development and progression of diabetic nephropathy, and support the further study of Gremlin as a therapeutic target in the treatment of DN. This work, then, has important implications for the future development of Gremlin inhibitory strategies.

## Materials and Methods

### Animal Model and Experimental Design

12-week-old male CD-1 mice (Charles River Laboratories, Vitalriver, Beijing, China) underwent uninephrectomy and were subsequently divided into three groups: a non-diabetic control group (N), a diabetic group administered a pBAsi mU6 Neo control plasmid (STZ), and a diabetic group administered a pBAsi mU6 Neo gremlin siRNA plasmid (Takara Bio Inc, Shiga, Japan) (Gremlin-si) (N = 18 per group). To induce diabetes, animals received a single dose of 150 mg/kg streptozotocin (Sigma, St. Louis, MO) in citrate buffer at pH 4.6 intravenously, two weeks after uninephrectomy. Hyperglycemia (>15 mmol/L) was confirmed 3 days after STZ administration. Plasmids were then delivered weekly by tail vein injection using the TransIT-EE Hydrodynamic Delivery System (Mirus Bio, Madison, USA). Animals were sacrificed at week-1, week-2 and week-12. At each time point, urine and blood samples were collected, and kidney tissues were harvested. All animal procedures were in accordance with the Guide for Care and Use of Laboratory Animals at the Department of Animal Resources, Hebei Medical University; the ethics committee approved this study (approval number: A-0709).

### Generation of pBAsi mU6 Neo Gremlin siRNA Plasmid

Three gremlin siRNA plasmids were constructed based on the U6 siRNA expression vector, pBAsi mU6 Neo (Takara, Mie, Japan), which includes a mouse U6 promoter and an amp-resistance gene. The following sets of sense and antisense oligonucleotides were annealed and ligated into the vector: sense oligo 1: 5′-GATCCGCACATCCGAAAGGAGGAATAGTGCTCCTGGTTGTTCCTCCTTTCGGATGTGCTTTTTTA- 3′, antisense oligo 1: 5′-AGCTTAAAAAAGCACATCCGAAAGGAGGAACAACCAGGAGCACTATTCCTCCTTTCGGATGTGCG-3′; sense oligo 2: 5′-GATCCGCCATCGACTTGGATTAAGTTAGTGCTCCTGGTTGACTTAATCCAAGTCGATGGCTTTTTTA- 3′, antisense oligo 2: 5′-AGCTTAAAAAAGCCATCGACTTGGATTAAGTCAACCAGGAGCACTAACTTAATCCAAGTCGATGGCG- 3′; sense oligo 3: 5′-GATCCGGATTTCACTTGAGAATGATAGTGCTCCTGGTTGTCATTCTCAAGTGAAATCCTTTTTTA- 3′, antisense oligo 3: 5′-AGCTTAAAAAAGGATTTCACTTGAGAATGACAACCAGGAGCACTATCATTCTCAAGTGAAATCCG- 3′.

### *In vivo* Delivery Method

To test the efficiency of the three pBAsi mU6 Neo gremlin siRNA plasmids, mouse mesangial cells cultured under high-glucose conditions were transfected with the plasmids, and the plasmids were also delivered into diabetic mice *in vivo*. Gremlin expression was evaluated by Western blot and immunohistochemistry. The most effective plasmid (oligo 1) was used for the study. Each diabetic animal received 30 µg of endotoxin-free plasmid DNA suspended in 2 ml of the TransIT-EE Hydrodynamic buffer weekly by tail vein injection according to the manufacturer's protocol.

### Light Microscopy

Kidneys were fixed in 4% paraformaldehyde and embedded in paraffin for light microscopy and immunohistochemistry. 2 µm sections were stained with Hematoxylin and Eosin (HE) and periodic acid-Schiff (PAS). The number of cells and diameter of glomeruli and tubules were quantitatively analyzed with the TD 2000 image pattern analysis system. Fifty glomeruli and 100 tubules for each animal were evaluated.

### *In vitro* Experiments

Mouse mesangial cells (MCs) were purchased from the American Type Culture Collection (Manassas, USA). Cells were grown in RPMI 1640 (Gibco) containing 5% FBS, penicillin (100 U/ml), streptomycin (100 µg/ml), and HEPES (14 mM) at 37°C and 5% CO_2_ -95% air. 2×10^6^ cells per well in 6-well culture plates or 2×10^5^ cells per each Lab-Tek16 chamber slide (Nalge Nunc International) were cultured without antibiotics for 24 hours. Then cells were transfected with pBAsi mU6 Neo gremlin siRNA plasmid or pBAsi mU6 Neo plasmid using lipofectamine 2000 reagent (Invitrogen). After 24 hours, cells were further cultured in DMEM containing high glucose (HG; 25 mM) or normal glucose (NG; 2.8 mM) for up to 48 hours. Cells in 6-well culture plates were collected for protein extraction. Cells on Lab-Tek16 chamber slides were fixed in 4% paraformaldehyde for immunochemistry, and culture medium was collected for Collagen IV measurement.

### RT-PCR

Total RNA was purified from mIMCD-3 cells with QIAzol Reagent (Qiagen). cDNA was synthesized from 2.5 g total RNA. The primer sequences are as follows: gremlin forward: 5′-GACAAGGCTCAGCACAATGA- 3′, gremlin reverse: 5′-AACTTCTTGGGCTTGCAGAA- 3′, BMP-7 forward: 5′-ACTCCTACATGAACGCCACC- 3′, BMP-7 reverse: 5′-GCTCAGGAGAGGTTGGTCTG- 3′, GAPDH forward: 5′- CCCACTAACATCAAATGGGG - 3′, GAPDH reverse: 5′- ATCCACAGTCTTCTG GGTGG - 3′. The relative abundance of mRNAs was standardized with GAPDH mRNA as the control.

### Western Blotting

30 µg of protein from each sample was subjected to SDS/PAGE under reducing conditions, and the gel proteins were electroblotted onto Hybond PVDF membrane (Amersham). Membranes were incubated with rabbit polyclonal anti- Gremlin, BMP-7, BMP-2, Smad5, Pho-Smad5, and TGF-beta antibodies (1∶500∼1∶1000, Santa Cruz) overnight, and then the membranes were incubated with anti-rabbit IgG conjugated to horseradish peroxidase (1∶20,000) at 37°C for 1 hour. After washing with PBST, the blots were incubated with ECL® Plus Western Blotting Detection Reagent (Amersham) and then exposed to X-ray film.

### Immunohistochemistry and Immunocytochemistry

The paraformaldehyde-fixed and paraffin-embedded kidney tissues were cut into sections of 4 µm thickness. After deparaffinization and rehydration, the slides were incubated with 3% H_2_O_2_ for 15 minutes at room temperature to block any intrinsic peroxidase activity and with 20% normal goat serum for 2 hours at 37°C to prevent non-specific binding of serum proteins. For immunohistochemistry, the tissues were then incubated sequentially with antibodies against PCNA or Gremlin (1∶100 or 1∶50 respectively, Santa Cruz) for 1 hour at 37°C, biotinylated anti-rabbit or anti-mouse IgG (1∶100; Gibco-BRL) for 20 min and streptavidin-peroxidase conjugate for 20 min. For immune-double staining, the tissues were incubated with a mixture of mouse anti-PCNA (1∶50) and rabbit anti-Gremlin (1∶50). Anti-PCNA antibodies were detected using goat anti-Mouse IgG-HRP with DAB reagent to produce brown staining. Anti-Gremlin antibodies were detected using goat anti-Rabbit IgG-AP with Fast-Red reagent to produce red staining.

### Gelatin Zymography

MMP-2 activity was determined by zymography by measuring gelatinolytic activity in culture media. Briefly, culture medium sampled after the desired incubation was centrifuged by 2000 rpm for 10 min. Protein concentration was determined by Bradford method. 40 µg of protein from each sample was applied to a 10% zymography gel and electrophoresed constantly at 90 mA for 60 min. Gels were firstly washed twice with washing buffer (2.5% Triton X-100,50 mmol/L Tris –HCl, 5 mmol/L CaCl2, 1 µmol/L ZnCl2, pH 7. 6) for 45 min, followed by a 42 hour incubation in a buffer containing 50 mmol/L Tris-HCl, 5 mmol/CaCl2, 1 µmol/L ZnCl2, 0. 02% Brij-35, pH 7.6. Gels were finally stained with Coomassie blue, and images were captured with a gel scanner. The clear zone on a dark background represented enzyme activity. Quantitation of bands was performed by densitometry.

### ELISA

Gremlin expression levels in culture medium were measured by a commercial ELISA kit (Adlitteram Diagnostic Laboratories, USA) according to the manufacturer's instructions. The absorbance was measured at 492 nm using a micro plate reader (Model 680, Bio-Rad). The results were expressed in nanograms per milliliter according to the calibration curve obtained with serial dilutions of a known quantity of Gremlin, and these were then normalized to the β-actin content of the corresponding tissues. The procedure was performed three times for each sample.

### Terminal Deoxynucleotidyl Transferase (TdT)-mediated dUTP Nick-end Labeling (TUNEL)

Measurement of apoptotic cells was performed using terminal deoxynucleotidyl transferase (TdT)-mediated dUTP nick-end labeling (TUNEL) with the *in situ* Apoptosis Detection Kit (Chemicon International, Temecula, CA, USA). Briefly, deparaffinized sections of mouse kidney were digested with proteinase K solution (Gibco BRL) (20 µg/ml) for 20 minutes at room temperature. Slides were rinsed in water and treated with 0.3% H_2_O_2_ for 10 minutes at room temperature. Test slides were incubated in terminal deoxytransferase (TdT) with biotin-dUTP for 1 hour at 37°C. Slides were washed in water, incubated with strepavidin-horseradish peroxidase complex for 30 minutes at room temperature, and detected with DAB (3-amino-9-ethylcarbazole) solution (Sigma) for 10 minutes. The numbers of TUNEL positive cells were counted in 50 glomeruli and in 10^4^ µm^2^ tubulointerstitial area.

### Immunoprecipitation

Mouse mesangial cells were lysed in RIPA buffer (20 mM Tris-HCl, pH 7.4, 100 mM NaCl, 1 mM CaCl_2_, 1 mM MgCl_2_, and 1% Triton X-100) with protease inhibitors. The lysates were centrifuged at 12,000 *g* for 30 minutes at 4°C, and the supernatants were incubated with preimmune sera and protein A-Sepharose (Amersham Biosciences, GE Healthcare, Uppsala, Sweden). Immunoprecipitations were performed by adding 10 µl of rabbit antibodies against mouse Gremlin or BMP-7 (1∶100, Santa Cruz) and 80 µl of protein-A Sepharose to 0.5 ml of supernatants. Normal rabbit IgG was used as a negative control. They were incubated overnight at 4°C on a rocking platform. The immunoprecipitated complexes were dissolved in a gel-loading buffer (50 mM Tris-HCl, pH 6.8, 2% SDS, 10% glycerol, 100 mM DTT, and 0.1% bromophenol blue), subjected to SDS/PAGE under reducing conditions, and electroblotted onto Hybond P PVDF membranes (Amersham Biosciences, Piscataway, NJ). The membranes were immunoblotted with specific antibodies against BMP-7 or Gremlin (1∶1000) overnight at 4°C and then with the secondary antibodies conjugated to horseradish peroxidase (1∶20000). Finally, the membranes were immersed in ECL Plus Western Blotting Detection Reagent (Amersham) and exposed to Hyperfilm ECL (Amersham).

### Statistical Evaluation

Data are presented as mean ± standard deviation (SD). Statistical analysis was performed by one-way ANOVA with Fisher t. P value of <0.05 was considered significant. The data were analyzed with Dr. SPSS II for Windows release 11.0.1J.

## References

[1] United States Renal Data System (1999). Excerpts from United States Renal Data System 1999 Annual Data Report.. Am J Kidney Dis.

[2] Bani-Hani AH, Campbell MT, Meldrum DR, Meldrum KK (2008). Cytokines in epithelial-mesenchymal transition: a new insight into obstructive nephropathy.. J Urol.

[3] Bottinger EP, Bitzer M (2002). TGF-beta signaling in renal disease.. J Am Soc Nephrol.

[4] Chen B, Athanasiou M, Gu Q, Blair DG (2002). Drm/Gremlin transcriptionally activates p21(Cip1) via a novel mechanism and inhibits neoplastic transformation.. Biochem Biophys Res Commun.

[5] Dolan V, Murphy M, Sadlier D, Lappin D, Doran P (2005). Expression of gremlin, a bone morphogenetic protein antagonist, in human diabetic nephropathy.. Am J Kidney Dis.

[6] Dronavalli S, Duka I, Bakris GL (2008). The pathogenesis of diabetic nephropathy.. Nat Clin Pract Endocrinol Metab.

[7] Fioretto P, Kim Y, Mauer M (1998). Diabetic nephropathy as a model of reversibility of established renal lesions.. Curr Opin Nephrol Hypertens.

[8] Fioretto P, Sutherland DE, Najafian B, Mauer M (2006). Remodeling of renal interstitial and tubular lesions in pancreas transplant recipients.. Kidney Int.

[9] Griffith DL, Keck PC, Sampath TK, Rueger DC, Carlson WD (1996). Three-dimensional structure of recombinant human osteogenic protein 1: structural paradigm for the transforming growth factor beta superfamily.. Proc Natl Acad Sci U S A.

[10] Helder MN, Ozkaynak E, Sampath KT, Luyten FP, Latin V (1995). Expression pattern of osteogenic protein-1 (bone morphogenetic protein-7) in human and mouse development.. J Histochem Cytochem.

[11] Hensey C, Dolan V, Brady HR (2002). The Xenopus pronephros as a model system for the study of kidney development and pathophysiology.. Nephrol Dial Transplant.

[12] Hruska KA, Guo G, Wozniak M, Martin D, Miller S (2000). Osteogenic protein-1 prevents renal fibrogenesis associated with ureteral obstruction.. Am J Physiol Renal Physiol.

[13] Kitten AM, Kreisberg JI, Olson MS (1999). Expression of osteogenic protein-1 mRNA in cultured kidney cells.. J Cell Physiol.

[14] Lappin DW, McMahon R, Murphy M, Brady HR (2002). Gremlin: an example of the re-emergence of developmental programmes in diabetic nephropathy.. Nephrol Dial Transplant.

[15] Maciel TT, Melo RS, Schor N, Campos AH (2008). Gremlin promotes vascular smooth muscle cell proliferation and migration.. J Mol Cell Cardiol.

[16] Marchant K (2008). Diabetes and chronic kidney disease: a complex combination.. Br J Nurs.

[17] Massague J (1990). The transforming growth factor-beta family.. Annu Rev Cell Biol.

[18] McMahon R, Murphy M, Clarkson M, Taal M, Mackenzie HS (2000). IHG-2, a mesangial cell gene induced by high glucose, is human gremlin. Regulation by extracellular glucose concentration, cyclic mechanical strain, and transforming growth factor-beta1.. J Biol Chem.

[19] Michos O, Goncalves A, Lopez-Rios J, Tiecke E, Naillat F (2007). Reduction of BMP4 activity by gremlin 1 enables ureteric bud outgrowth and GDNF/WNT11 feedback signalling during kidney branching morphogenesis.. Development.

[20] Morrissey J, Hruska K, Guo G, Wang S, Chen Q (2002). Bone morphogenetic protein-7 improves renal fibrosis and accelerates the return of renal function.. J Am Soc Nephrol.

[21] Murphy M, Godson C, Cannon S, Kato S, Mackenzie HS (1999). Suppression subtractive hybridization identifies high glucose levels as a stimulus for expression of connective tissue growth factor and other genes in human mesangial cells.. J Biol Chem.

[22] Nishimura R, Hata K, Ikeda F, Matsubara T, Yamashita K (2003). The role of Smads in BMP signaling.. Front Biosci.

[23] Sugimoto H, Grahovac G, Zeisberg M, Kalluri R (2007). Renal fibrosis and glomerulosclerosis in a new mouse model of diabetic nephropathy and its regression by bone morphogenic protein-7 and advanced glycation end product inhibitors.. Diabetes.

[24] Nohe A, Hassel S, Ehrlich M, Neubauer F, Sebald W (2002). The mode of bone morphogenetic protein (BMP) receptor oligomerization determines different BMP-2 signaling pathways.. J Biol Chem.

[25] Otani H, Otsuka F, Inagaki K, Takeda M, Miyoshi T (2007). Antagonistic effects of bone morphogenetic protein-4 and -7 on renal mesangial cell proliferation induced by aldosterone through MAPK activation.. Am J Physiol Renal Physiol.

[26] Ozkaynak E, Schnegelsberg PN, Oppermann H (1991). Murine osteogenic protein (OP-1): high levels of mRNA in kidney.. Biochem Biophys Res Commun.

[27] Petrovic MG, Korosec P, Kosnik M, Osredkar J, Hawlina M (2008). Local and genetic determinants of vascular endothelial growth factor expression in advanced proliferative diabetic retinopathy.. Mol Vis.

[28] Preisig P (1999). A cell cycle-dependent mechanism of renal tubule epithelial cell hypertrophy.. Kidney Int.

[29] Vukicevic S, Kopp JB, Luyten FP, Sampath TK (1996). Induction of nephrogenic mesenchyme by osteogenic protein 1 (bone morphogenetic protein 7).. Proc Natl Acad Sci U S A.

[30] Vukicevic S, Latin V, Chen P, Batorsky R, Reddi AH (1994). Localization of osteogenic protein-1 (bone morphogenetic protein-7) during human embryonic development: high affinity binding to basement membranes.. Biochem Biophys Res Commun.

[31] Wang S, Chen Q, Simon TC, Strebeck F, Chaudhary L (2003). Bone morphogenic protein-7 (BMP-7), a novel therapy for diabetic nephropathy.. Kidney Int.

[32] Wang S, de Caestecker M, Kopp J, Mitu G, Lapage J (2006). Renal bone morphogenetic protein-7 protects against diabetic nephropathy.. J Am Soc Nephrol.

[33] Wang S, Hirschberg R (2003). BMP7 antagonizes TGF-beta -dependent fibrogenesis in mesangial cells.. Am J Physiol Renal Physiol.

[34] Sneddon JB, Zhen HH, Montgomery K, van de Rijn M, Tward AD (2006). Bone morphogenetic protein antagonist gremlin 1 is widely expressed by cancer-associated stromal cells and can promote tumor cell proliferation.. Proc Natl Acad Sci U S A.

[35] Namkoong H, Shin SM, Kim HK, Ha SA, Cho GW (2006). The bone morphogenetic protein antagonist gremlin 1 is overexpressed in human cancers and interacts with YWHAH protein.. BMC Cancer.

[36] Pavel E, Zhao W, Powell KA, Weinstein M, Kirschner LS (2007). Analysis of a new allele of limb deformity (ld) reveals tissue- and age-specific transcriptional effects of the Ld Global Control Region.. Int J Dev Biol.

